# New Class of Efficient T_2_ Magnetic Resonance Imaging Contrast Agent: Carbon-Coated Paramagnetic Dysprosium Oxide Nanoparticles

**DOI:** 10.3390/ph13100312

**Published:** 2020-10-15

**Authors:** Huan Yue, Ji Ae Park, Son Long Ho, Mohammad Yaseen Ahmad, Hyunsil Cha, Shuwen Liu, Tirusew Tegafaw, Shanti Marasini, Adibehalsadat Ghazanfari, Soyeon Kim, Kwon Seok Chae, Yongmin Chang, Gang Ho Lee

**Affiliations:** 1 Department of Chemistry, Department of Nanoscience and Nanotechnology (DNN), College of Natural Sciences, Kyungpook National University (KNU), Taegu 41566, Korea; 20100819@hanmail.net (H.Y.); sonlongh@gmail.com (S.L.H.); yaseen.knu@gmail.com (M.Y.A.); liushuwen0701@gmail.com (S.L.); tegafawtirusew@yahoo.com (T.T.); shantimarasini.sm@gmail.com (S.M.); adibeh.ghazanfari@gmail.com (A.G.); 2 Division of RI-Convergence Research, Korea Institute of Radiological & Medical Sciences (KIRAMS), Seoul 01817, Korea; jpark@kirams.re.kr; 3 Department of Molecular Medicine and Medical & Biological Engineering, DNN, School of Medicine, KNU and Hospital, Taegu 41566, Korea; hscha1002@daum.net (H.C.); hoooot111@knu.ac.kr (S.K.); 4 Department of Biology Education, DNN, Teachers’ College, KNU, Taegu 41566, Korea; kschae@knu.ac.kr

**Keywords:** dysprosium oxide nanoparticle, carbon coating, efficient contrast agent, T_2_ magnetic resonance imaging

## Abstract

Nanoparticles are considered potential candidates for a new class of magnetic resonance imaging (MRI) contrast agents. Negative MRI contrast agents require high magnetic moments. However, if nanoparticles can exclusively induce transverse water proton spin relaxation with negligible induction of longitudinal water proton spin relaxation, they may provide negative contrast MR images despite having low magnetic moments, thus acting as an efficient T_2_ MRI contrast agent. In this study, carbon-coated paramagnetic dysprosium oxide (DYO@C) nanoparticles (core = DYO = Dy_x_O_y_; shell = carbon) were synthesized to explore their potential as an efficient T_2_ MRI contrast agent at 3.0 T MR field. Since the core DYO nanoparticles have an appreciable (but not high) magnetic moment that arises from fast 4f-electrons of Dy(III) (^6^H_15/2_), the DYO@C nanoparticles exhibited an appreciable transverse water proton spin relaxivity (r_2_) with a negligible longitudinal water proton spin relaxivity (r_1_). Consequently, they acted as a very efficient T_2_ MRI contrast agent, as proven from negative contrast enhancements seen in the in vivo T_2_ MR images.

## 1. Introduction

Nanotechnology and nanomaterials may provide a breakthrough in future medicine science [1,2]. Nanoparticles have tremendous potential for application in various medical fields owing to their unique and excellent properties, which are better than those of atomic, molecular, and bulk materials [3,4,5,6]. They may be applied as advanced negative (T_2_) magnetic resonance imaging (MRI) contrast agents to elevate negative contrast differentials between normal and abnormal tissues [7,8,9]; this effect cannot be obtained using molecular agents because molecules do not have sufficient magnetic moments. Since contrast agents are generally more accumulated in abnormal tissues than in normal ones, they may be used to sensitively diagnose abnormal tissues such as cancer cells at an early stage through contrast enhancements [10,11,12].

At present, Gd(III)-chelates are the most popular contrast agents because of their kinetic stability, biocompatibility, and renal excretion ability [13,14]. On the other hand, the carboxydextran-coated superparamagnetic iron oxide (SPIO) nanoparticles are the only commercially available negative (T_2_) MRI contrast agent [15]. However, the SPIO nanoparticles are generally limited to liver imaging because of their large particle diameters. For example, Resovist, a commercial SPIO-based contrast agent, is coated with dextran and has multiple SPIOs with a diameter of 4.2 nm at the core. It has a hydrodynamic diameter of 60 nm and is used for liver imaging [15]. Therefore, new ultrasmall nanoparticles that can be used for various organs and that possess renal excretion ability should be developed as a new class of T_2_ MRI contrast agents.

Nanoparticles composed of lanthanide (Ln) elements such as Dy^3+^ (^6^H_15/2_), Ho^2+^ (^5^I_8_), Tb^3+^ (^7^F_6_), Er^3+^ (^4^I_15/2_), and Tm^3+^ (^3^H_6_) with magnetic moment components originating from fast 4f-electron orbital motions induce negligible longitudinal water proton spin relaxation because the fast electrons are far from the slow proton spin motions [14]. In addition, transverse relaxation can occur without causing longitudinal relaxation; however, the converse is not true [16]. Therefore, such nanoparticles can exclusively induce transverse water proton spin relaxation. Hence, they may provide negative contrasts in the in vivo T_2_ MR images even though their transverse relaxation induction is not strong, but merely appreciable; thus, these nanoparticles can function as an efficient T_2_ MRI contrast agent.

So far, few studies have explored Ln_2_O_3_ nanoparticles. Most of them were relaxometric studies [17,18,19], and a few included in vivo MRI studies [20,21,22]. For in vivo applications, nanoparticles should be coated with biocompatible and hydrophilic ligands. In this study, ultrasmall dysprosium oxide (= DYO = Dy_x_O_y_) nanoparticles were synthesized by a polyol method; then, they were coated with carbon in aqueous media by dehydrating dextrose under basic conditions. Carbon as one of the most common elements in living objects is suitable for biomedical applications [23,24,25]. In addition, carbon materials have fluorescent properties in the visible region owing to the numerous conjugated C=C bonds, thus allowing optical imaging [25]. The synthesized DYO@C core–shell nanoparticles (core = DYO; shell = carbon) were stable in a colloidal form because of the numerous hydroxyl groups on the carbon surfaces. These groups originated from dextrose. The DYO@C nanoparticles were characterized by various experimental techniques to investigate their potential as a new class of efficient T_2_ MRI contrast agent in 3.0 T MR field.

## 2. Results

### 2.1. Size, Colloidal Stability, and Crystallinity of DYO@C Nanoparticles

**Figure 1:**
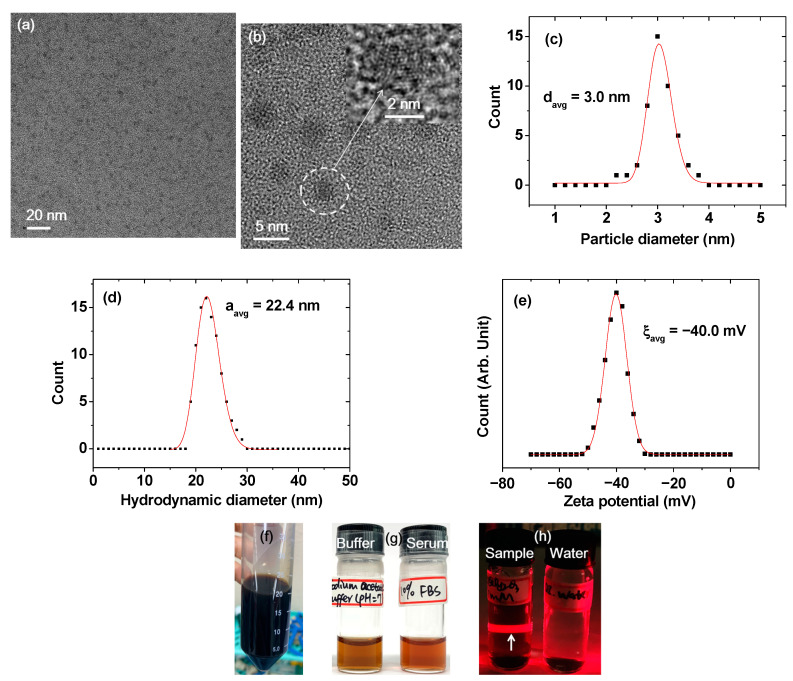
Various characterization results of the DYO@C nanoparticles: (**a**) TEM image at the 20-nm scale; (**b**) HRTEM image at the 5-nm scale (inset is a magnified image of the dotted circle at the 2-nm scale); (**c**) plot of particle diameter distribution and a log-normal function fit to obtain d_avg_; (**d**) plot of hydrodynamic diameter distribution and a log-normal function fit to obtain a_avg_; (**e**) plot of zeta potential and a Gaussian function fit to obtain ξ_avg_; (**f**) concentrated aqueous solution sample (18 mM Dy); (**g**) photographs of the nanoparticle suspension samples in a 10% FBS in RPMI1640 medium (right) and a sodium acetate buffer (pH = 7) solution (left; 1.8 mM Dy) showing no nanoparticle precipitation for 10 days; and (**h**) Tyndall effect showing laser light scattering (indicated with an arrow) due to nanoparticle suspension (left) and the reference triple-distilled water, which did not show laser light scattering (right).

The DYO@C nanoparticles (DYO = Dy_x_O_y_) were ultrasmall and nearly monodisperse in the particle diameter, as shown in their transmission electron microscope (TEM) images (Figure 1a,b). The lattice fringes of the core DYO nanoparticle on the dark carbon-coating layer can be seen under the magnified high-resolution TEM (HRTEM) image, thus proving the core–shell structure of the DYO@C nanoparticles (inset in Figure 1b). The average particle diameter (d_avg_) was estimated to be 3.0 nm from a log-normal function fit to the observed particle diameter distribution (Figure 1c and Table 1). The average hydrodynamic diameter (a_avg_) was estimated to be 22.4 nm from a log-normal function fit to the observed dynamic light scattering (DLS) pattern (Figure 1d and Table 1). This large hydrodynamic diameter is attributed to the abundant OH groups on the carbon-coating surface layer covering the nanoparticle; these OH groups attracted numerous water molecules. This structure explains the observed good colloidal stability of the carbon-coated nanoparticles in an aqueous solution. The colloidal stability was also confirmed from the high zeta potential (ξ_avg_ = −40.0 mV) of the carbon-coated nanoparticles in an aqueous solution (Figure 1e). Similar large hydrodynamic diameters and consequently, good colloidal stabilities were observed in many polymer-coated nanoparticles [26,27,28]; these previous studies support our result. In general, hydrodynamic diameter is due to both surface-coating materials and hydrated water molecules. From the difference between the hydrodynamic diameter and the core diameter measured from HRTEM imaging, and considering that numerous hydrated water molecules contribute to the hydrodynamic diameter, it is expected that the upper bound value of the coating layer thickness will be 9–10 nm. The carbon-coated nanoparticles were not precipitated at all after synthesis (>1 year): a photograph of a concentrated aqueous nanoparticle solution sample (18 mM Dy) is shown in Figure 1f. In addition, no precipitation of the DYO@C nanoparticles in a 10% fetal bovine serum (FBS) in RPMI1640 medium and a sodium acetate buffer solution (pH = 7.0; 1.8 mM Dy) was observed for 10 days, thereby indicating good colloidal stability (Figure 1g). The laser light scattering (or the Tyndall effect) was only observed for the nanoparticle suspension sample (left photograph in Figure 1h; in this case, a diluted solution sample was used to visually observe the laser path, as indicated with an arrow), but not in the reference triple-distilled water (right photograph in Figure 1h). Thus, the colloidal dispersion of the carbon-coated nanoparticles in an aqueous solution was confirmed.

**Figure 2:**
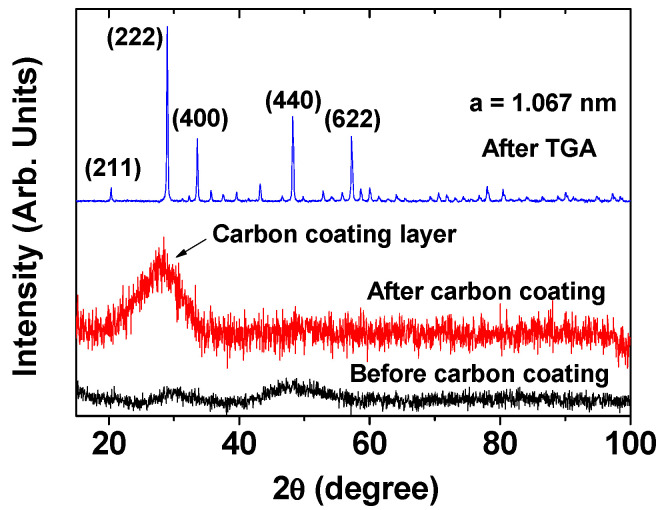
XRD patterns of the DYO nanoparticles before carbon coating (bottom spectrum), and DYO@C nanoparticles before TGA (middle spectrum) and after TGA (top spectrum). All peaks after TGA could be assigned with (hkl) Miller indices corresponding to cubic Dy_2_O_3_ and only the strong peaks were assigned.

Before carbon coating, the DYO nanoparticles displayed a broad and amorphous X-ray diffraction (XRD) pattern due to their incomplete crystallization arising from their ultrasmall nanoparticle size [29] (bottom pattern in Figure 2). Here, the DYO nanoparticle is assigned as DyxOy because its structure is unknown (i.e., amorphous). The DYO@C nanoparticles showed an additional broad peak at 2θ = 20–33° (centered at 28°) arising from the carbon-coating layer, likely corresponding to the C (002) peak of amorphous carbon [30,31] (middle pattern in Figure 2). After a thermogravimetric analysis (TGA) up to 900 ℃ of the powder sample, however, only the sharp peaks of cubic Dy_2_O_3_ appeared due to the crystallization accompanying crystal growth and the combustion removal of the carbon-coating layer (top pattern in Figure 2). The estimated cell constant (10.67 Å) of the Dy_2_O_3_ nanoparticles obtained after TGA was consistent with the reported value of 10.670 Å [32].

### 2.2. Surface-Coating Amount and Surface-Coating Structure

**Figure 3:**
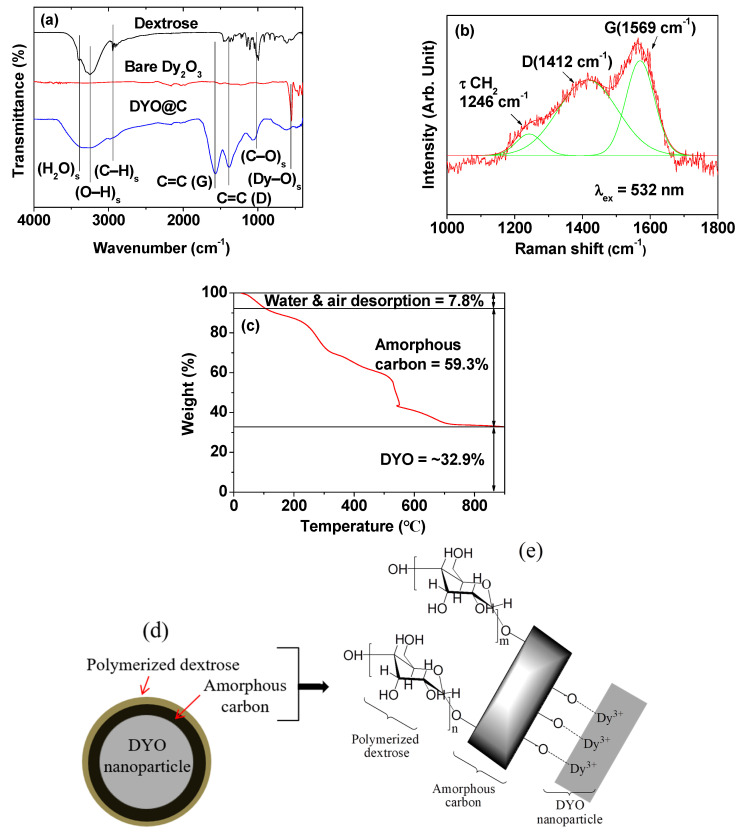
(**a**) FT-IR absorption spectra of the free dextrose (top spectrum), bare Dy_2_O_3_ nanoparticles (middle spectrum obtained after TGA), and DYO@C nanoparticles (bottom spectrum); (**b**) Raman spectrum of the DYO@C nanoparticles (excitation laser wavelength, λ_ex_ = 532 nm); (**c**) TGA curve of the DYO@C nanoparticles; proposed (**d**) carbon-coating structure of the DYO@C nanoparticles and (**e**) carbon-coating layer structure.

Two additional peaks, corresponding to the G- and D-bands of C=C stretching were observed at 1568 and 1384 cm^−1^ [33,34,35], respectively, in the Fourier transform-infrared (FT-IR) absorption spectrum of the powder sample (bottom spectrum in Figure 3a). These peaks were absent in the spectrum of free dextrose (top spectrum in Figure 3a) because of no C=C bond in free dextrose [36], thus confirming the carbon coating on the nanoparticle surface. The Dy-O stretching peak was observed at 550 cm^−1^ in the FT-IR absorption spectra of both the sample and the bare Dy_2_O_3_ nanoparticles, which were obtained after TGA (middle spectrum in Figure 3a), confirming the presence of DYO nanoparticles in the sample. Raman spectrum also confirmed G-and D-bands, which appeared at 1569 and 1412 cm^−1^ [37], respectively (Figure 3b). The D-band in both FT-IR absorption and Raman spectra was overlapped with CH_2_ scissoring (δ), wagging (ω), and twisting (τ) vibrations, which appear in the region of 1270–1460 cm^−1^ [37]. The strong O-H stretching peak at 3240 cm^−1^ in the spectrum of the sample confirmed the existence of a large number of OH groups in the sample. This O–H stretching peak did not result from water H–O–H stretching, for which the peak appeared at 3390 cm^−1^, as shown in Figure 3a. The O–H, C–O (at 1065 cm^−1^), and C–H (at 2970 cm^−1^) stretching peaks in the FT-IR absorption spectrum of the sample indicate that the carbon-coating layer was not completely carbonated. The carbon-coating layer seems to have polymerized dextrose layers on its surface, as reported elsewhere [38]. 

The amounts of carbon coating and core DYO nanoparticles were estimated to be 59.3 and 32.9 wt%, respectively, from a TGA curve (Figure 3c and Table 1) after considering the initial mass drop of 7.8% up to 105 °C resulting from water and air desorption from the powder sample. In addition, the surface-coating amount was estimated to be 63.32% (Table 1) from the elemental analysis (EA) of the powder sample by summing the obtained C/H/O wt% of 31.82/3.55/27.95 (=1.51/2.02/1.00 in a mole), which was roughly consistent with the TGA results.

The carbonation percentage of the carbon-coating layer was estimated to be 33.8% (=(0.51/1.51) × 100) using the enhanced carbon content from dextrose (i.e., 1.0 (dextrose) → 1.51 (carbon-coating layer); the C/H/O mole ratio of dextrose (C_6_H_12_O_6_) is 1.0/2.0/1.0). Therefore, the remaining 66.2% of the carbon-coating layer corresponded to the polymerized dextrose layer. Since carbon nanoparticles are formed through dextrose polymerization and terminates with hydrophilic polymerized dextrose at the carbon nanoparticle surface [38], it seems that carbon coating starts with dextrose polymerization on the DYO nanoparticle surface. Then, the polymerized dextrose becomes amorphous carbon, which is made of almost randomly oriented aromatic carbon sheets [30] and terminates with polymerized dextrose, similar to the carbon nanoparticle formation [38]. The observed good colloidal stability of the DYO@C nanoparticles confirms that the hydrophobic amorphous carbon is terminated with hydrophilic polymerized dextrose. Therefore, a carbon-coating structure of the DYO@C nanoparticles is proposed as shown in Figure 3d,e. As shown in Figure 3e, the amorphous carbon was conjugated to Dy^3+^ ions on the DYO nanoparticle surface through oxygen ions, and the presence of numerous OH groups of the polymerized dextrose on the carbon-coating surface imparted good colloidal stability to the DYO@C nanoparticles in an aqueous solution.

**Figure 4:**
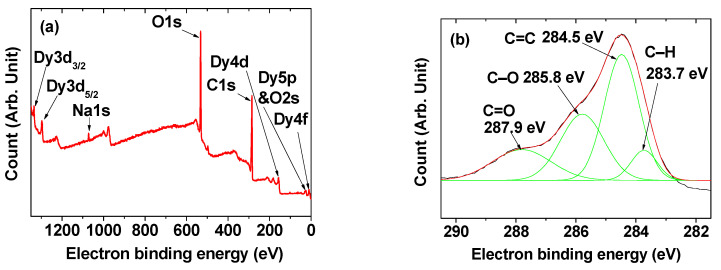
XPS spectra of DYO@C nanoparticles. (**a**) Whole range scan showing C, O, Na, and Dy elements in the nanoparticle sample and (**b**) carbon peak composed of four different carbons.

The XPS spectrum showed C, O, Na, and Dy in the nanoparticle sample (Figure 4a). Here, Na resulted from NaOH used in the synthesis and came from charge balance of the DYO@C nanoparticles with negative zeta potential. In addition, the XPS spectrum indicated the presence of various carbons such as C–H (from polymerized dextrose), C=C (from amorphous carbon), C–O (from polymerized dextrose), and C=O (from amorphous carbon; Figure 4b), supporting the presence of amorphous carbon and polymerized dextrose in the carbon-coating layer. The observed electron binding energies (EBEs) of all elements are provided in Table 2 and consistent with literature values [39,40,41,42].

### 2.3. Magnetic Properties

**Figure 5:**
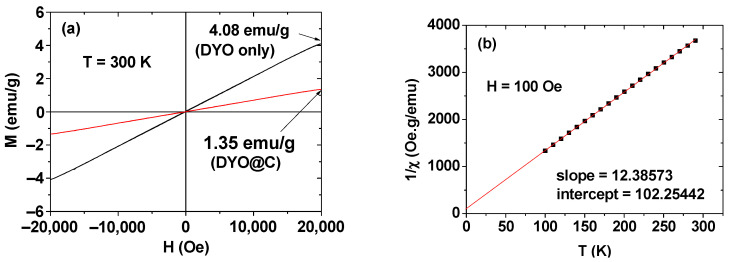
Magnetic properties of DYO@C nanoparticles. (**a**) M–H curves before and after mass correction at 300 K and (**b**) plot of the Curie–Weiss law using the mass-corrected M. The mass correction of M was done using the net mass of DYO nanoparticles obtained from TGA.

The magnetization (M) versus applied field (H) curves (i.e., M–H curves) of DYO@C nanoparticles before and after mass correction at 300 K are shown in Figure 5a. The mass correction of M was performed using the net mass of DYO nanoparticles obtained from TGA. The mass effect of the nearly non-magnetic carbon-coating layer on M can be clearly observed; it is seen that the M value of the sample decreased because of the carbon-coating layer with a mass wt% of 59.3 according to the TGA results. The M–H curves showed that the core DYO nanoparticles were paramagnetic (i.e., zero coercivity, zero remanence, no saturation magnetization, and no hysteresis in the M–H curve) as in the case of bulk material [43,44]. The M value increased with increasing H and reached 4.08 emu/g at 2.0 T. This appreciable M value originated from the fast 4f-electrons of Dy^3+^ [45] and corresponded to the exclusive induction of transverse water proton spin relaxation.

For paramagnetic materials, the Curie–Weiss law (i.e., χ = M/H = C/(T–T_c_) in which χ is the magnetic susceptibility, C is the Curie constant, and T_c_ is the Curie temperature) can be applied [46]. As shown in Figure 5b, a linear plot was obtained, confirming the paramagnetism of the core DYO nanoparticles. From the plot, C = 0.08074 emuK/gOe was estimated, which is a small value because the core DYO nanoparticles are paramagnetic. 

### 2.4. In Vitro Cytotoxicity Results

**Figure 6:**
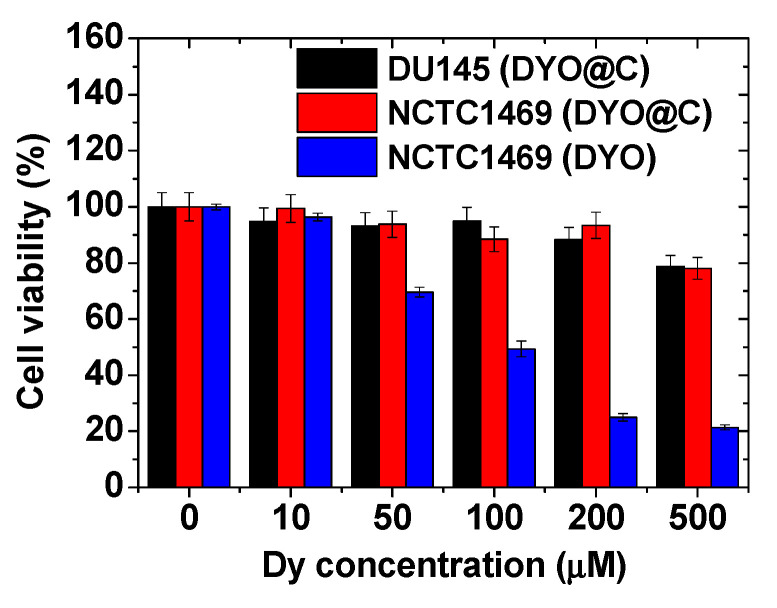
Cell viabilities of DYO@C nanoparticles in NCTC1469 and DU145 cells and those of DYO nanoparticles before carbon coating in NCTC1469 cells.

As shown in Figure 6, the cell viabilities of human prostate cancer (DU145) and normal mouse hepatocyte (NCTC1469) cells treated with the aqueous solution sample were good (>80%) up to 500 μM Dy, supporting good biocompatibility of the DYO@C nanoparticles. However, the DYO nanoparticles before carbon coating exhibited high cellular toxicities at Dy-concentration greater than 50 μM Dy, confirming the necessity of carbon coating on the DYO nanoparticle surface for biomedical applications.

### 2.5. Longitudinal (r_1_) and Transverse (r_2_) Water Proton Spin Relaxivities

**Figure 7:**
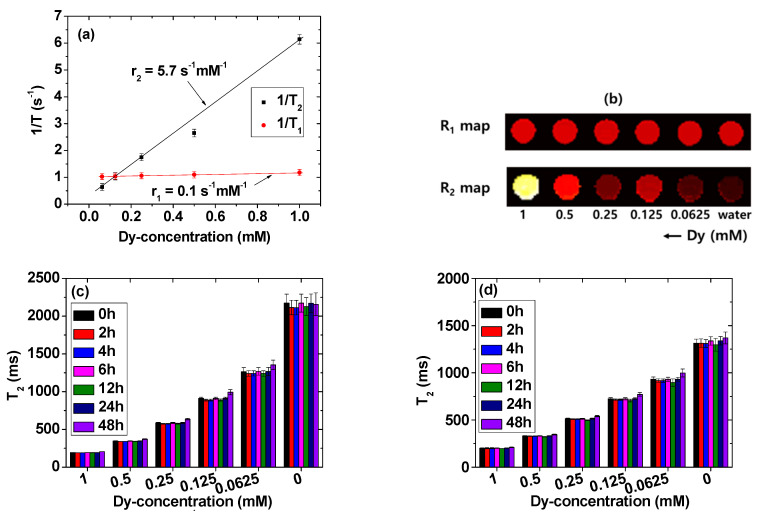
Relaxometric properties of the DYO@C nanoparticles in an aqueous solution. (**a**) Plots of 1/T_1_ and 1/T_2_ as a function of Dy-concentration in the 3.0 T MR field and the slopes correspond to r_1_ and r_2_ values, respectively; (**b**) R_1_ and R_2_ map images as a function of Dy-concentration, showing dose-dependent contrast enhancements in the R_2_ map images but not in the R_1_ map images; and T_2_ relaxation times overtime in (**c**) triple-distilled water and (**d**) a 10% FBS in RPMI1640 medium as a function of Dy-concentration.

The r_1_ and r_2_ values of the solution sample were estimated to be 0.1 and 5.7 s^−1^mM^−1^ from the slopes of the inverse plots of longitudinal (T_1_) and transverse (T_2_) water proton spin relaxation times versus Dy-concentration, respectively (Figure 7a). The appreciable r_2_ and negligible r_1_ values (r_2_/r_1_ = 57) suggest that the DYO@C nanoparticles are solely devoted to inducing transverse water proton spin relaxation. Hence, they should negligibly induce longitudinal water proton spin relaxation. This was confirmed from the clear dose-dependent contrast enhancements in the R_2_ map images; in contrast, the R_1_ map images showed hardly any dose-dependent contrast enhancement (Figure 7b). This observation was further confirmed based on the in vivo T_2_ MR images as discussed below.

T_2_ relaxation times were also measured overtime (0, 2, 4, 6, 12, 24, and 24 h) in triple-distilled water (Figure 7c) and a 10% FBS in RPMI1640 medium (Figure 7d) as a function of Dy-concentration. T_2_ relaxation times were constant overtime within an experimental error limit due to good colloidal stability of the DYO@C nanoparticles in an aqueous solution and a 10% FBS in RPMI1640 medium. 

### 2.6. In Vivo T_2_ MR Images

**Figure 8:**
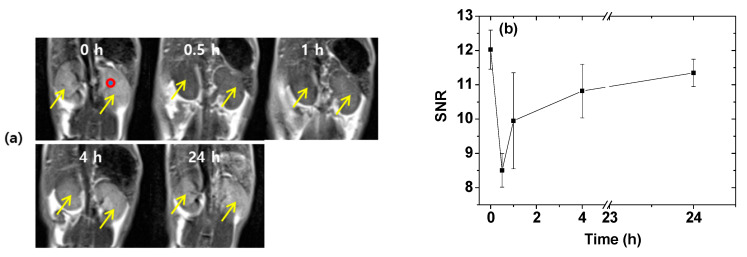
In vivo MRI results of the aqueous DYO@C nanoparticle solution sample. (**a**) In vivo coronal T_2_ MR images of the mice kidneys (indicated with arrows) as a function of time (a small circle indicated a region-of-interest (ROI) used for the signal-to-noise ratio (SNR) plot) and (**b**) SNR plot of ROI as a function of time. Here, 0 h indicates preadministration, and the remaining time points indicate follow-up as a function of time after administration of the aqueous solution sample into mice tail veins. Four mice were used.

The application of DYO@C nanoparticles as an efficient T_2_ MRI contrast agent was demonstrated from in vivo T_2_ MR images of mice. Negative (i.e., darkened) contrast enhancements in the kidneys were clearly observed after intravenous administration of the aqueous nanoparticle suspension sample into mice tails (Figure 8a). The negative contrasts weakened with time (i.e., the signal-to-noise ratio (SNR) increased with time) and returned to the original contrast. To quantitatively measure the time evolution of the contrast changes, the SNR of a region-of-interest (ROI) as indicated by a small circle in the preadministration MR image (labeled as 0 h), was plotted as a function of time (Figure 8b). The plot showed that the negative contrast enhancements reached a maximum (or a minimum SNR) at 30 min after administration and then, decreased thereafter (or the SNR increased thereafter), finally reaching the initial preadministration value. However, in the liver, no noticeable negative contrast enhancements were observed at the same points after administration. This is likely because of the rapid excretion of the DYO@C nanoparticles from the liver within 30 min after administration. The SNR behavior in the kidneys was somewhat similar to that of the commercial molecular contrast agents [13,14]; this behavior is attributed to the renal excretion of the nanoparticles as observed for various ultrasmall nanoparticles in previous studies [47,48,49]. The observed appreciable negative contrast enhancement even at an appreciable (but not high) r_2_ value was due to a negligible r_1_ value (i.e., a very large r_2_/r_1_ ratio). The results prove that the DYO@C nanoparticles should function as an efficient T_2_ MRI contrast agent. 

### 2.7. Optical Properties: Ultraviolet (UV)-Visible Absorption and Photoluminescent (PL) Spectra

**Figure 9:**
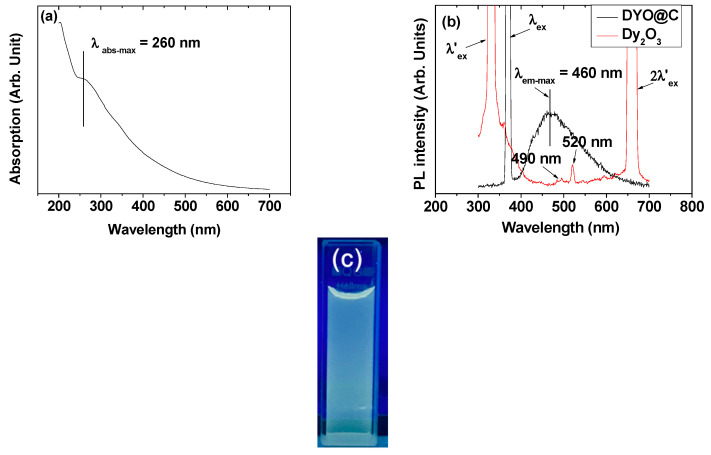
(**a**) UV-visible absorption spectrum of the aqueous DYO@C nanoparticle solution sample; (**b**) photoluminescent (PL) spectra of the aqueous DYO@C and Dy_2_O_3_ nanoparticle solution samples (λ_ex_ = 370 nm, λ’_ex_ = 330 nm, 490 nm = Dy: ^4^F_9/2_ → ^6^H_15/2_, and 520 nm = Dy: ^4^I_15/2_ → ^6^H_13/2_); and (**c**) photograph of the solution sample under 365 nm UV irradiation, showing blue-green fluorescence.

The amorphous carbon absorbs and emits visible photons because of the presence of conjugated C=C bonds [24,25]. These were confirmed from the absorption band at λ_abs-max_ = 260 nm in the UV-visible absorption spectrum (Figure 9a) and the emission band at λ_em-max_ = 460 nm in the photoluminescent (PL) spectrum (excitation wavelength, λ_ex_ = 370 nm; Figure 9b) of an aqueous solution sample, as observed in the amorphous carbon nanoparticle solution sample [24]. A PL spectrum (λ_ex_ = 330 nm) of the aqueous Dy_2_O_3_ nanoparticle solution sample, which was prepared by dispersing TGA-treated Dy_2_O_3_ nanoparticles in triple-distilled water, was also taken for reference (Figure 9b), showing weak Dy-transitions at 490 nm (^4^F_9/2_ → ^6^H_15/2_) and 520 nm (^4^I_15/2_ → ^6^H_13/2_) [50,51]. Therefore, most of the PL in the DYO@C nanoparticle sample solution was due to the amorphous carbon-coating layer. Here, the emission peak at 520 nm was assigned based on energetic consideration between electronic energy levels [51] below the λ_ex_ = 330 nm. The quantum yield (QY) of the DYO@C nanoparticle solution sample was estimated to be 6.5% using fluorescein with a QY value of 95% as the reference [24]. This estimated value was consistent with that of amorphous carbon nanoparticle solution sample [24]. Under 365-nm UV irradiation, the aqueous nanoparticle suspension sample exhibited blue-green fluorescence (Figure 9c), corresponding to the emission range observed in the PL spectrum. The fluorescence in the visible region will be useful for fluorescence imaging, which was confirmed by cell imaging using carbon nanoparticles in previous studies [25]. 

## 3. Discussion

Both the r_2_ value and r_2_/r_1_ ratio should be high for T_2_ MRI contrast agents. From the data provided in Table 1, r_2_ value (=5.7 s^−1^mM^−1^) was just appreciable but r_2_/r_1_ (=57) was very high due to a negligible r_1_ value (=0.1 s^−1^mM^−1^) under a 3.0 T MR field. Since r_2_ value was not high, the DYO@C nanoparticles will not be a strong T_2_ MRI contrast agent under clinical MR fields (i.e., 1.5–3.0 T). However, the DYO@C nanoparticles could act as a very efficient T_2_ MRI contrast agent under the above conditions, as observed in this study. That is, there were appreciable negative contrast enhancements in the in vivo T_2_ MR images of mice under a 3.0 T MR field (Figure 8a).

The colloidal stability, biocompatibility, and renal excretion of nanoparticles were essential for in vivo applications. The high zeta potential (Figure 1d) and no precipitation (Figure 1e–g) of the DYO@C nanoparticles indicate that they have excellent colloidal stability. They were nearly non-toxic as indicated by the in vitro cellular cytotoxicity results (Figure 6). They were removed by renal excretion as indicated in the in vivo T_2_ MR images (Figure 8a,b), showing their suitability for in vivo applications.

Compared to the SPIO nanoparticles with a very high r_2_ value and an appreciable r_2_/r_1_ ratio (Table 3) [15,52,53], the DYO@C nanoparticles are less powerful because of their smaller r_2_ value, but more efficient because of their higher r_2_/r_1_ ratio under clinical MR fields (i.e., 1.5–3.0 T). Under clinical MR fields, the SPIO-based nanoparticles are generally used as liver imaging contrast agents due to their large particle diameters or aggregations [15,53]. However, the DYO@C nanoparticles may not be limited to the liver imaging but also applicable to various organ and tissue imagings because of their renal excretion ability resulting from their ultrasmall core particle size with no aggregation [47,48,49]. In addition, the M value of the DYO@C nanoparticles increases with increasing H (Figure 5a), and consequently, their r_2_ value increases with increasing MR field because r_2_ value is proportional to M^2^ [17,18]. In fact, very high r_2_ values were observed in high MR fields [18]. This implies that the DYO@C nanoparticles will be a very powerful T_2_ MRI contrast agent under high MR fields such as 9.4 T, which results from the combined effects of their high r_2_ value under high MR fields and very high r_2_/r_1_ ratio. On the other hand, for gadolinium oxide (Gd_2_O_3_) nanoparticles and Gd(III)-DTPA, r_2_/r_1_ ratio is close to one [54,55], due to slow s-state 4f-electron motions of Gd^3+^ (^7/2^S), which match well with slow proton spin motions. Under these conditions, they can efficiently induce longitudinal water proton spin relaxation. Therefore, they are considered positive (T_1_) MRI contrast agents.

## 4. Materials and Methods

### 4.1. Materials

Dy(NO_3_)_3_⋅5H_2_O (99.9%), NaOH (>99.9%), triethylene glycol (TEG) (99%), dextrose (C_6_H_12_O_6_) (>99.5%), dialysis bags (MWCO = 2000 amu), sodium acetate buffer solution (3.0 M, pH = 7.0), FBS, and RPMI1640 medium were purchased from Sigma-Aldrich, St. Louis, MO, USA, and used as received. Ethanol (99%, Duksan, Korea) was used for the initial washing of the nanoparticles. Triple-distilled water was used for the final washing of the nanoparticles and for the preparation of the nanoparticle suspension sample.

### 4.2. Synthesis of DYO@C Nanoparticles

**Figure 10:**
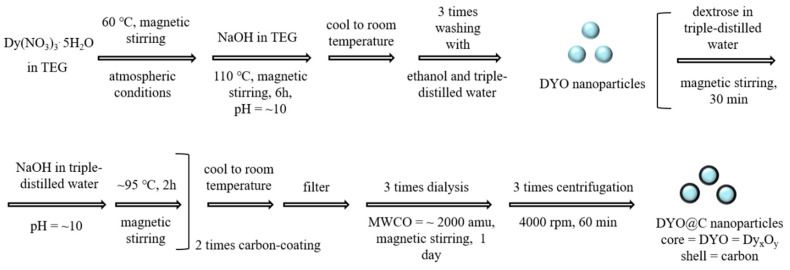
Synthesis of DYO@C core–shell nanoparticles (core = DYO = Dy_x_O_y_ and shell = carbon): the first step is the synthesis of DYO nanoparticles in TEG and the second step is the carbon coating on the DYO nanoparticle surface by dehydrating dextrose in a basic aqueous medium.

The DYO@C core–shell nanoparticles were synthesized in two steps (Figure 10): first, DYO nanoparticles were synthesized in TEG and then, DYO nanoparticles were coated with carbon using dextrose as a carbon source in a basic aqueous solution. Four solutions were prepared: (1) a precursor solution made of 1.0 mmol of Dy(NO_3_)_3_⋅5H_2_O in 20 mL of TEG in a 100 mL three-necked-flask, (2) a NaOH solution made of 4 mmol of NaOH in 10 mL of TEG in a 50 mL beaker, (3) a dextrose solution made of 1.0 mmol of dextrose in 10 mL of triple-distilled water in a 100 mL three-necked-flask, and (4) a NaOH solution made of 4 mmol of NaOH in 10 mL of triple-distilled water in a 50 mL beaker. Solution-1 was magnetically stirred at 60 °C under atmospheric conditions until Dy(NO_3_)_3_⋅5H_2_O dissolved in TEG. Solution-2 was slowly added to solution-1 until the pH of the solution reached 10. The mixed solution was slowly heated to 110 °C with magnetic stirring for 6 h. For temperature control, the three-necked-flask was suspended in a silicone oil bath placed on a hot plate. The solution was cooled to room temperature, transferred to a 1.0 L beaker, diluted with 500 mL of ethanol, magnetically stirred for 30 min, and then kept in a refrigerator (3 °C) until the DYO nanoparticles settled to the bottom of the breaker. The top transparent solution was decanted and the remaining DYO nanoparticle solution was washed with ethanol again by the same process: this process was performed thrice. To remove ethanol from the DYO nanoparticles, the solution was washed with triple-distilled water thrice following the same process as above. The washed DYO nanoparticles were added to solution-3 and the mixed solution was magnetically stirred for 30 min. For carbon coating, solution-4 was added to the above solution until the pH of the solution reached 10 and the mixed solution was magnetically stirred at 95 °C until the solution became black: this carbon-coating process was performed twice. The solution was cooled to room temperature, filtered with a Whatman filter paper (Sigma-Aldrich, St. Louis, MO, USA), transferred to a dialysis bag, and dialyzed against 1.0 L of triple-distilled water for three days with magnetic stirring to remove free dextrose and unreacted NaOH; waste water was replaced with fresh triple-distilled water at one day intervals. To remove free carbon nanoparticles from the product nanoparticles, the product solution was centrifuged at 4000 rpm for 60 min (VS-4000N, Vision Scientific Co., LTD, Limassol, Cyprus). The supernatant top solution was removed and the remaining product nanoparticles that were precipitated at the bottom of the centrifugation tube were redispersed in triple-distilled water. This centrifugation process was performed thrice. The obtained solution sample was divided into two equal volume parts. One part was used for preparing a nanoparticle suspension sample in triple-distilled water. The remaining was transformed into a powder by freeze-drying it in vacuum for various characterizations. Stability of the prepared solution sample was confirmed from dialysis (Mw = 2000 amu) in triple-distilled water for one week: no carbon and Dy^3+^ in the waste water were detected from the EA and ICPAES, respectively.

### 4.3. Characterizations

An HRTEM (Titan G2 ChemiSTEM CS Probe, FEI) operated at 200 kV was used to measure the particle diameter (d) of the DYO@C nanoparticles. For measurements, a drop of the diluted nanoparticle suspension in triple-distilled water was put onto a carbon film supported by a 200-mesh copper grid (PELCO no.160, Ted Pella, Inc., Redding, CA, USA) placed on a filter paper using a micropipette (2–20 μL, Eppendorf), and then dried in air at room temperature. The Dy-concentration in an aqueous nanoparticle suspension sample was measured using an inductively coupled plasma-atomic emission spectrometer (ICP-AES; Optima 7300DV and Avio500, Perkin Elmer, Waltham, MA, USA). The nanoparticle suspension sample was pretreated with acids to completely dissolve the nanoparticles into metal ions (Dy^3+^) in the solution before measurements. A multipurpose XRD spectrometer (X’PERT PRO MRD, Philips, The Netherlands) with unfiltered CuKα radiation (λ = 0.154184 Å) was used to measure the crystal structure of the powder sample before and after TGA. The scan range was 2θ = 15–100° and the scan step was 2θ = 0.03°. A DLS particle size analyzer (UPA-150, Microtrac) was used to measure the hydrodynamic diameter (a) of the DYO@C nanoparticles dispersed in an aqueous solution using a nanoparticle suspension sample (<0.1 mM Dy). The zeta potentials (Zetasizer Nano ZS, Malvern, Malvern, UK) were measured using the nanoparticle suspension sample (<0.1 mM Dy). An FT-IR absorption spectrometer (Galaxy 7020A, Mattson Instruments, Inc., Madison, WI, USA) and a Raman spectrometer (invia Reflex, Renishaw, Charfield, UK) were used to investigate the carbon-coating on the nanoparticle surfaces by recording the FT-IR absorption and Raman spectra, respectively. A pellet of the powder sample in KBr was prepared for measurements. To estimate the surface-coating amount of the carbon on the nanoparticle surfaces, a TGA instrument (SDT-Q600, TA Instruments, New Castle, DE, USA) was used to record the TGA curve using a powder sample between room temperature and 900 °C under air flow. The average surface-coating amount was estimated from the mass loss after taking into account the initial mass loss between room temperature and 105 °C due to water and air desorption. The amount of DYO nanoparticles was approximately estimated from the remaining mass. After TGA, the powder sample was collected and subjected to XRD analysis. Through the EA (Flash 2000, ThermoFisher, Waltham, MA, USA), the surface-coating amount and the surface composition (C/H/O) were estimated using a powder sample. An X-ray photoelectron spectrometer (XPS; NEXSA, ThermoFisher, Waltham, MA, USA) was used to characterize the surface composition of the DYO@C nanoparticles using the powder sample. For measurements, the nanoparticle powder sample was put onto a carbon tape (1 cm × 1 cm). A vibrating sample magnetometer (VSM; 7407-S, Lake Shore Cryotronics Inc., Westerville, OH, USA) was used to characterize the magnetic properties of the powder sample by recording an M–H curve (−2.0 T ≤ H ≤ 2.0 T) at 300 K and an M-T curve (100 ≤ T ≤ 300 K) at H = 100 Oe. The measurement was carried out using a powder sample (20−30 mg). To obtain a net M value of the core DYO nanoparticles, the measured M was mass-corrected by using the net mass of the DYO nanoparticles (i.e., only the mass of DYO nanoparticles without carbon in the sample) as obtained from TGA. Since carbon materials absorb and emit visible photons, a UV-visible absorption spectrum (Cary-Series, Agilent Technologies, Santa Clara, CA, USA) and a PL spectrum (Cary Eclipse, Agilent Technologies, Santa Clara, CA, USA) were recorded using an aqueous nanoparticle suspension sample. The nanoparticle suspension sample was filled into a quartz cuvette with two optically clear sides (Sigma-Aldrich, 3 mL) for UV-visible absorption spectral measurements and into a quartz cuvette with four optically clear sides (Sigma-Aldrich, 3 mL) for PL spectral measurements.

### 4.4. In Vitro Cell Viability Measurements

The in vitro cytotoxicity of the aqueous nanoparticle suspension sample was measured using a luminescent cell viability assay (CellTiter-Glo, Promega, Madison, WI, USA). Adenosine triphosphate was quantified using a luminometer (Victor 3, Perkin Elmer, Waltham, MA, USA). DU145 and NCTC1469 cell lines were used. The cells were seeded onto a 24-well cell culture plate and incubated for 24 h (5 × 10^4^ cell density, 500 μL cells per well, 5% CO_2_, and 37 °C). Five test nanoparticle suspension samples (10, 50, 100, 200, and 500 μM Gd) were prepared by diluting the original concentrated nanoparticle suspension sample with a sterile phosphate buffer saline solution. Each test nanoparticle suspension sample (2 μL) was dropped onto the cells. The treated cells were incubated for 48 h. The cell viabilities were measured thrice and normalized with respect to that of the control cells (i.e., untreated cells with 0.0 M Dy).

### 4.5. Measurements of Water Proton Spin Relaxation Times and Map Images

A 3.0 T MRI scanner (MAGNETOM Trio Tim, Siemens, Munchen, Bayern, Germany) was used to measure the T_1_ and T_2_ water proton spin relaxation times, and the R_1_ and R_2_ water proton spin relaxation map images at 22 °C. Various aqueous nanoparticle suspension samples (1.0, 0.5, 0.25, 0.125, 0.0625, and 0.0 mM Dy) were prepared via dilution of the original concentrated sample with triple-distilled water. These diluted samples were used to measure the T_1_ and T_2_ relaxation times and R_1_ and R_2_ map images. T_1_ relaxation time measurements were performed using an inversion recovery method. The Carr–Purcell–Meiboom–Gill pulse sequence for multiple spin-echo measurements was used to obtain T_2_ relaxation times. Then, the r_1_ and r_2_ water proton spin relaxivities of the nanoparticle suspension sample were estimated from the slopes of plots of 1/T_1_ and 1/T_2_ versus the Dy-concentration, respectively.

### 4.6. In Vivo T_2_ MR Image Measurements

In vivo MRI studies using mice were approved by the animal research committee of the KIRAMS and were performed in accordance with its rules and guidelines. In vivo T_2_ MR images were acquired using a 3.0 T MRI scanner. Four mice were used. For imaging, C57BL/6 mice (30 g) were anesthetized with 1.5% isoflurane in oxygen. Measurements were made before and after administration of the nanoparticle suspension sample into mice tail veins. The administration dose was typically 0.1 mmol Dy per kg. After measurements, the mice were revived from anesthesia and placed in a cage with free access to food and water. During measurements, the temperature of the mice was maintained at 37 °C using a warm water blanket. The parameters used for measurements were as follows: external MR field = 3.0 T; temperature = 37 °C; number of acquisitions = 4; field of view (FOV) = 9 mm; phase FOV = 0.5; matrix size = 256 × 192; slice thickness = 1 mm; spacing gap = 0.5 mm (coronal); pixel bandwidth = 15.63 Hz; repetition time = 500 ms; and echo time = 13 ms.

## 5. Conclusions

DYO@C core–shell nanoparticles (core = DYO = Dy_x_O_y_; shell = carbon) were synthesized and their potential as a new class of T_2_ MRI contrast agent was investigated in a 3.0 T MR field. The in vitro cellular cytotoxicity assay showed that they were nearly non-toxic. They were stable in the colloidal form in an aqueous solution due to the presence of numerous hydroxyl groups on the carbon-coating layer. The r_2_ value of the nanoparticles was only 5.7 s^−1^mM^−1^, but their r_2_/r_1_ (=57) was very high. Therefore, the DYO@C nanoparticles acted as a very efficient T_2_ MRI contrast agent. That is, they clearly showed negative contrast enhancements in the in vivo T_2_ MR images of the mice kidneys after intravenous administration. In addition, the fluorescence of the carbon-coating layer in the visible region may perhaps make the nanoparticles useful as a multimodal imaging agent.

## Figures and Tables

**Table 1:** Summarized properties of DYO@C nanoparticles.

d_avg_^1^ (nm)	a_av_^2^ (nm)	ξ_avg_^3^ (mV)	Surface Coating Amount (wt%)		M ^4^ at 2.0 T (emu/g)	r_1_^5^ (s^−1^mM^−1^)	r_2_^6^ (s^−1^mM^−1^)	r_2_/r_1_	λ_abs-max_^7^ (nm)	λ_em__-max_^8^ (nm)	η ^9^ (%)
			TGA	EA							
3.0 ± 0.1	22.4 ± 0.1	−40.0 ± 0.2	59.3	63.32	4.08	0.1	5.7	57	260	460	6.5

^1^ average particle diameter; ^2^ average hydrodynamic diameter; ^3^ average zeta potential; ^4^ net magnetization of DYO nanoparticles; ^5^ longitudinal water proton spin relaxivity (22 °C, 3.0 T); ^6^ transverse water proton spin relaxivity (22 °C, 3.0 T); ^7^ maximum absorption wavelength; ^8^ maximum emission wavelength; ^9^ quantum yield.

**Table 2:** Electron binding energies (EBEs) of the elements observed in XPS spectra of the DYO@C nanoparticles.

Element	Orbital	Observed EBE (eV)	Literature	Ref.
C	1s, C=O	287.9	288.3	[42]
	1s, C–O	285.8	285.6	[42]
	1s, C=C	284.5	284.3, 284.5, 284.4	[40,41,42]
	1s, C–H	283.7	281.8	[42]
O	1s	531.6	531	[39]
	2s	25.0	23	[39]
Na	1s	1072.0	1072.1	[39]
Dy	3d_3/2_	1334.5	1333	[39]
	3d_5/2_	1296.4	1296	[39]
	4d	155.6	152	[39]
	5p	25.0	23	[39]
	4f	10.0	8	[39]

**Table 3:** r_1_ and r_2_ values of various nanoparticles and a chelate.

Nanoparticle	d_avg_ (nm)	a_avg_ (nm)	Coating Material	r_1_ (s^−1^mM^−1^)	r_2_ (s^−1^mM^−1^)	r_2_/r_1_	H (tesla)	T (℃)	Medium	Ref.
DYO	3.0	22.4	Carbon	0.1	5.7	57	3.0	22	Water	This work
Fe_3_O_4_/γ-Fe_2_O_3_^1^	4.2	60	Dextran 40000	19.4	185.8	9.6	0.47	40	Plasma	[15]
Fe_3_O_4_^2^	4.9	21	Dextran T-10	22.7	53.1	2.3	0.47	39	Water	[52,53]
Gd_2_O_3_	3.1	18.9	Carbon	16.3	24.1	1.5	3.0	22	Water	[54]
Gd(III)-DTPA ^3^	-	-	-	4.5	5.7	1.3	0.47	39	Water	[55]

^1^ Ferucarbotran (Resovist): clinically approved T_2_ MRI contrast agent for the liver (Schering AG, Germany) and multiple SPIO nanoparticles are coated with dextran 40,000; ^2^ Ferumoxtran-10 (AMI-227): clinically approved T_2_ MRI contrast agent for the lymph node (Guerbet, France); ^3^ DTPA = diethylenetriaminepenta-acetic acid.
